# Impact of Length or Relevance of Questionnaires on Attrition in Online Trials: Randomized Controlled Trial

**DOI:** 10.2196/jmir.1733

**Published:** 2011-11-18

**Authors:** Jim McCambridge, Eleftheria Kalaitzaki, Ian R White, Zarnie Khadjesari, Elizabeth Murray, Stuart Linke, Simon G Thompson, Christine Godfrey, Paul Wallace

**Affiliations:** ^1^Faculty of Public Health & PolicyLondon School of Hygiene & Tropical MedicineLondonUnited Kingdom; ^2^MRC General Practice Research FrameworkLondonUnited Kingdom; ^3^MRC Biostatistics UnitInstitute of Public HealthCambridgeUnited Kingdom; ^4^E-health UnitResearch Department of Primary Care and Population HealthUniversity College LondonLondonUnited Kingdom; ^5^Department of Health Sciences and HYMSUniversity of YorkYorkUnited Kingdom

**Keywords:** Attrition, retention, missing data, response rates, alcohol, online

## Abstract

**Background:**

There has been limited study of factors influencing response rates and attrition in online research. Online experiments were nested within the pilot (study 1, n = 3780) and main trial (study 2, n = 2667) phases of an evaluation of a Web-based intervention for hazardous drinkers: the Down Your Drink randomized controlled trial (DYD-RCT).

**Objectives:**

The objective was to determine whether differences in the length and relevance of questionnaires can impact upon loss to follow-up in online trials.

**Methods:**

A randomized controlled trial design was used. All participants who consented to enter DYD-RCT and completed the primary outcome questionnaires were randomized to complete one of four secondary outcome questionnaires at baseline and at follow-up. These questionnaires varied in length (additional 23 or 34 versus 10 items) and relevance (alcohol problems versus mental health). The outcome measure was the proportion of participants who completed follow-up at each of two follow-up intervals: study 1 after 1 and 3 months and study 2 after 3 and 12 months.

**Results:**

At all four follow-up intervals there were no significant effects of additional questionnaire length on follow-up. Randomization to the less relevant questionnaire resulted in significantly lower rates of follow-up in two of the four assessments made (absolute difference of 4%, 95% confidence interval [CI] 0%-8%, in both study 1 after 1 month and in study 2 after 12 months). A post hoc pooled analysis across all four follow-up intervals found this effect of marginal statistical significance (unadjusted difference, 3%, range 1%-5%, *P* = .01; difference adjusted for prespecified covariates, 3%, range 0%-5%, *P* = .05).

**Conclusions:**

Apparently minor differences in study design decisions may have a measurable impact on attrition in trials. Further investigation is warranted of the impact of the relevance of outcome measures on follow-up rates and, more broadly, of the consequences of what we ask participants to do when we invite them to take part in research studies.

**Trial registration:**

ISRCTN Register 31070347; http://www.controlled-trials.com/ISRCTN31070347/31070347 Archived by WebCite at (http://www.webcitation.org/62cpeyYaY)

## Introduction

A large multidisciplinary experimental literature has developed over many decades in which a wide range of methods to increase response rates in postal surveys have been evaluated [1]. Edwards and colleagues included 481 trials in their updated systematic review of this literature, which includes both postal and electronic surveys [2]. Among the methods identified to be effective in postal surveys are using shorter questionnaires (pooled odds ratios [ORs] for 56 trials for responding to shorter vs longer questionnaires = 1.64, 95% confidence interval [CI] 1.43-1.87) and asking more relevant questions (pooled ORs for 3 trials = 2.00, 95% CI 1.32-3.04). The findings from 32 randomized controlled trials of electronic surveys have been broadly similar to those obtained for postal surveys [2].

It is unclear to what extent findings on methods effective in enhancing response rates in surveys can be applied to studies involving follow-up. Attrition prevention may involve issues that are different from those concerned with maximizing survey response rates because being interviewed or providing questionnaire data some time after study entry is likely to be influenced by the history of study involvement and the demands it makes upon the participant.

There is not, however, a clear evidence base on effective methods to prevent loss to follow-up specifically in the contexts of cohort studies and trials. A 2007 systematic review of retention strategies in health care research found no studies that “explicitly compared the effectiveness of different retention strategies” [3]. A 2009 systematic review of drug trials for weight loss found that the number of attendances for research purposes made no difference to attrition [4]. In the same year, a meta-analysis found a range of study design characteristics to influence attrition in trials of antidepressants among older people [5].

It is important to minimize attrition in these types of studies, as participants lost to follow-up may have characteristics different from participants retained by the study, thus potentially introducing bias. Attrition is particularly problematic for online trials as it is usually substantial [6-7] and may differ between randomized groups, thus engendering additional difficulties in interpretation of study findings [8]. Attrition in online trials is also well known to be a more complex phenomenon than in conventional trials [6-7]. The online context permits intervention nonusage, which can be very high. This is often closely related to loss to follow-up for research purposes. It is this latter form of attrition that is the subject of the present study.

In planning the Down your Drink randomized controlled trial (DYD-RCT), as an attrition reduction measure, we decided to reduce the assessment burden by randomly allocating participants to complete only one of four secondary outcome questionnaires rather than all four [9]. Although this decision sacrificed statistical power to detect effects on these particular outcomes, it added to the capacity to detect effects on the primary outcome to the extent that reducing the overall assessment burden enhances follow-up rates [6]. In so doing, we created the opportunity for a methodological experiment as these secondary questionnaires varied in length and relevance, both characteristics known to influence survey response rates [2]. We tested two hypotheses: (1)longer questionnaires (23 or 34 versus 10 items included in secondary outcome measures) will produce lower rates of follow-up and (2)more relevant questionnaires (defined as assessing alcohol problems rather than mental health) will produce higher rates of follow-up.

## Methods

### Study Procedures and Participants

The methodological studies reported here were embedded in DYD-RCT, a large trial of an online intervention to help hazardous drinkers reduce their alcohol consumption [9]. The parent study included a pilot phase followed by the main trial. The pilot phase involved an unusually large sample, greater than that required for the main trial. We undertook the present methodological experiment in both phases of the parent trial with one alteration made to the design of the second study (see below). We also explored the effects of incentives on follow-up rates in randomized studies, which have been separately reported and do not influence the findings of the present study.

Potential study participants originally accessed a webpage inviting them to “find out if you are drinking too much,” and were then asked to complete a brief 3-item screening test. If eligible, they were invited to take part and given access to a consent page after an information page. Eligible participants were people drinking potentially unhealthy levels of alcohol who were also willing to consider changing their behaviour. After a password had been created and email details validated, participants completed the EQ-5D, a well known brief health-related quality of life measure, and calculated their past week alcohol consumption based on specific alcohol brands and volumes. This is a complex task requiring time and effort varying with amount and patterns of drinking. Participants subsequently answered two questions on confidence and intentions before arriving at a *final questionnaire* prior to being told their parent trial group allocation. Without their knowledge, participants had been randomly allocated to one of four different questionnaires (described below) to be completed as this final questionnaire*.* Participants were thus blinded to the conduct of this study.

All participants thus completed common trial entry and baseline research assessments with the sole difference between the study groups being the secondary outcome measure (ie, the final questionnaire) to which they had been randomly allocated. In both phases, randomization was performed by a computer-generated randomization procedure. Randomization could not be subverted, therefore, by the study team, and allocation was fully concealed. Randomization to a particular secondary outcome measure applied to baseline and both subsequent follow-ups. Participants were thus offered the same secondary outcome questionnaire at all three time points. Randomization was performed separately and independently from randomization to intervention and control conditions in the parent trial [9]. The numbers of participants in the present study slightly exceed those in the parent trial as some participants completed the first randomization to secondary outcome questionnaire and did not complete the subsequent randomization to parent trial study condition. 

Participants were sent email requests for follow-up data in the pilot phase after 1 and 3 months (study 1) and in the main trial phase after 3 and 12 months (study 2). Data collected at follow-up consisted of past week alcohol consumption, the EQ-5D, single-item measures of confidence and intention, and the same secondary outcome measure completed at baseline. Up to three reminders were sent at 7-day intervals to nonresponders, with the final reminder containing a request for participants to tell us their past week alcohol consumption only. Ethical approval was obtained from University College London ethics committee.

### Outcomes and Measures

In both studies the sole outcome evaluated here was the proportion of participants who responded, that is, completed the primary outcome questionnaires within 40 days of the email request. The three alcohol problems measures used in both studies were the Alcohol Use Disorders Identification Test (AUDIT), which is the screening test for hazardous and harmful drinking recommended by the World Health Organization [10]; the Leeds Dependence Questionnaire (LDQ), which assesses severity of alcohol dependence [11]; and the Alcohol Problems Questionnaire (APQ), which assesses problems other than dependence [12]. We used the core 23 items of the APQ. The AUDIT and LDQ both comprise 10 items. These instruments all require the respondent to report whether drinking is responsible for a range of difficulties they may experience. Mental health was assessed with different versions of the same instrument: the full 23 or 34 item CORE-OM (Clinical Outcomes in Routine Evaluation-Outcome Measure) in study 1 and the newer, briefer 10-item CORE-10 in study 2 [13-14]. This instrument makes no reference to alcohol.

### Statistical Methods

The analyses followed an analysis plan that was written before the relevant data were analyzed. The main analyses compared the proportion responding at each time point between those randomized to longer (APQ and CORE-OM) and shorter (AUDIT, LDQ, and CORE-10) questionnaires and between those randomized to questionnaires relevant to alcohol problems (AUDIT, LDQ, and APQ) and questionnaires less relevant to alcohol problems, being concerned with mental health (CORE-OM and CORE-10). Comparisons were expressed as differences in proportions (risk differences) for interpretability and odds ratios for comparability with other literature. 

As a sensitivity analysis, we used logistic regression to adjust for the following baseline variables that were previously found to be predictive of attrition: parent trial group allocation (DYD or comparator), age, gender, educational attainment (degree level or not), ethnicity (white British or other), whether an address was given at study entry, health state, baseline weekly alcohol consumption, and intention (scored 1 to 5). Pooled analyses (that were not prespecified) combined all four follow-up assessments and allowed for the correlation between the two follow-up assessments for the same person using generalized estimating equations adjusting for study and occasion [15]. Prespecified subgroup analyses explored, using interaction tests on both scales, whether any effects differed by gender, parent trial group allocation, educational attainment (university or college degree obtained or not), and baseline weekly alcohol consumption. Baseline weekly alcohol consumption was dichotomized so that women drinking under 35 United Kingdom (UK) units of alcohol and men drinking under 50 UK units of alcohol in the past week were classified as lighter drinkers and those drinking above these levels were deemed heavier drinkers with 1 UK unit being equivalent to 8 grams of ethanol. 

## Results

Randomization was successful in creating groups equivalent for comparison purposes (Table 1). The total number who consented to participate in the parent trial between February 2007 through August 2008 was 8285 (4957 in study 1 and 3328 in study 2). Of these, 1838 did not complete earlier recruitment steps prior to being randomized to secondary outcome questionnaires, resulting in 6447 study participants for whom results are reported in Table 1. The follow-up rates in groups randomized to the four secondary outcome measures at all four follow-up intervals are presented in Table 2.

**Table 1:** Baseline characteristics of groups randomized to four secondary outcome measures

	Secondary Outcome Measure					
Baseline Characteristic	AUDIT	LDQ	APQ	CORE-OM^a^	CORE-10^a^	CORE-OM or CORE-10^a^
Number	1614	1607	1613	945	668	1613
Female (%)	58	55	57	55	60	57
Intervention (%)	50	49	50	53	50	52
Heavy drinking (%)	62	62	61	59	62	61
Educated to degree level (%)	50	53	52	50	48	49
White British (%)	84	84	85	84	84	84
Provided postal address (%)	36	34	35	35	35	35
Intentions score, median (interquartile range)	4 (2)	4 (2)	4 (2)	4 (2)	4 (2)	4 (2)
Age, mean (SD)	37.9 (10.7)	37.9 (10.6)	38.0 (10.9)	37.0 (11.0)	37.4 (10.6)	37.2 (10.9)
Health state, mean (SD)	67.4 (22.6)	66.9 (27.1)	67.4 (23.2)	70.8 (46.0)	66.2 (22.9)	68.9 (38.3)
Past week alcohol consumption (UK units), mean (SD)	56.2 (37.9)	57.2 (37.4)	56.8 (40.4)	54.8 (36.3)	56.6 (37.7)	55.5 (36.9)

^a^CORE-OM was used in study 1, CORE-10, in study 2

**Table 2:** Follow-up rates in groups randomized to four secondary outcome measures

		Secondary Outcome Measure			
Follow-up Period		AUDIT	LDQ	APQ	CORE-OM or CORE-10^a^ [1]
**Study 1**					
	1 month	497/949 (52%)	552/939 (59%)	529/947 (56%)	489/945 (52%)
	3 months	376/949 (40%)	403/939 (43%)	414/947 (44%)	378/945 (40%)
**Study 2**					
	3 months	337/665 (51%)	316/668 (47%)	316/666 (47%)	308/668 (46%)
	12 months	222/665 (33%)	225/668 (34%)	213/666 (32%)	194/668 (29%)

^a^CORE-OM in study 1, CORE-10 in study 2

Shown in Table 3 are comparisons of the follow-up rates between groups randomized to longer (23 or 34 items) and shorter (10 items) secondary outcome measures. Note that the sample sizes are similar in study 1 as there were two questionnaires in each category and dissimilar in study 2 where there was only one longer questionnaire (APQ) and three shorter ones. There is no evidence of any difference in attrition due to additional questionnaire length, and the 95% confidence interval suggests that any difference is no more than 2 percentage points.

**Table 3:** Follow-up rates in those allocated to longer and shorter secondary outcome questionnaires

		Questionnaire Length				
		Longer	Shorter	Longer vs Shorter		
Follow-up Period				Difference	Odds Ratio	*P Value*
**Study 1**						
	1 month	1018/1892 (54%)	1049/1888 (56%)	−0.02% (−0.05% to 0.01%)	0.93 (0.82-1.06)	.28
	3 months	792/1892 (42%)	779/1888 (41%)	0.01% (−0.03% to 0.04%)	1.03 (0.90-1.17)	.71
**Study 2**						
	3 months	316/666 (47%)	961/2001 (48%)	−0.01% (−0.05% to 0.04%)	0.98 (0.82-1.16)	.80
	12 months	213/666 (32%)	641/2001 (32%)	−0.00% (−0.04% to 0.04%)	1.00 (0.83-1.20)	.98
	Pooled analysis of both studies at all four follow-up intervals			−0.00% (−0.03% to 0.02%)	0.98 (0.89-1.07)	.67
	Pooled analysis adjusted for covariates			−0.00% (−0.02% to 0.02%)	0.98 (0.90-1.08)	.75

Data comparing follow-up rates in groups randomized to the three measures of alcohol problems with those randomized to the mental health measure in both study 1 and study 2 are presented in Table 4. The post hoc pooled analysis identifies relevance to alcohol problems to be associated with a 3 percentage point increase in response rate, a result that was clearly statistically significant on unadjusted analysis but only of borderline statistical significance in the sensitivity analysis adjusting for baseline covariates.

Subgroup analyses by the four prespecified covariates identified no strong evidence of effect modification. All *P* values for interaction terms were in excess of .05 whether analyzed separately by study and time (as was prespecified) or pooled over studies and times.

**Table 4:** Follow-up rates in those allocated to more and less relevant (alcohol problems and mental health respectively) secondary outcome questionnaires

		Questionnaire Focus				
		Alcohol Problems	Mental Health	Alcohol Problems vs Mental Health		
Follow-up Period				Difference	Odds Ratio	*P Value*
**Study 1**						
	1 month	1578/2835 (56%)	489/945 (52%)	0.04% (0.00%-0.08%)	1.17 (1.01-1.36)	.04
	3 months	1193/2835 (42%)	378/945 (40%)	0.02% (−0.02% to 0.06%)	1.09 (0.94-1.27)	.26
**Study 2**						
	3 months	969/1999 (48%)	308/668 (46%)	0.02% (−0.02% to 0.07%)	1.10 (0.92-1.31)	.29
	12 months	660/1999 (33%)	194/668 (29%)	0.04% (−0.00% to 0.08%)	1.20 (0.99-1.46)	.05
	Pooled analysis of both studies at all four follow-up intervals			0.03% (0.01%-0.05%)	1.14 (1.03-1.25)	.01
	Pooled analysis adjusted for covariates			0.03% (0.00%-0.05%)	1.11 (1.00-1.22)	.05

## Discussion 

Allocating participants to longer secondary outcome questionnaires did not lead to lower rates of follow-up when comparing 23 or 34 versus 10 items in addition to completion of primary outcome measures and associated trial entry procedures. More precisely, inspection of the confidence intervals indicates that secondary outcome questionnaire length does not reduce follow-up rates by more than approximately 2%. More relevant questionnaires assessing alcohol problems rather than mental health did produce higher rates of follow-up though the difference was small, being not greater than 5%, and the statistical significance was doubtful in the sensitivity analysis.These two main findings will first be considered separately.

### Questionnaire Length

The unusual decision to randomize to secondary outcome measures was made to minimize attrition, both because we were persuaded by existing high quality evidence of the effects of questionnaire length on response rate and also because attrition was correctly anticipated as a formidable challenge in a trial undertaken completely online. We did not, however, investigate overall assessment burden, which could have been done by making a randomized comparison between the total burden, that is, completion of all secondary outcome measures, which is standard practice, versus one only. This would have required a comparison that assigned a large proportion of participants to a condition expected to be unfavorable to retention in the trial, and, therefore, we chose not to do this. This original study design decision is reemphasised here because of the implications for the interpretation of study findings.

We found that asking participants to answer an additional 23 or 34 questions rather than an additional 10 questions did not influence the likelihood of retention in the study. The unit of analysis in previous postal studies has been the number of pages per questionnaire [2] rather than the number of items per questionnaire, as was used in the present study. In both the previously cited review and in a related systematic review and meta-regression study, Edwards and colleagues identified significant unexplained heterogeneity in the effects of questionnaire length [16]. Effects were greatest when postcards were compared with conventional questionnaires. In six trials comparing one page against either two or three pages, no differences in response rate were observed. Only in the five trials in which a one-page questionnaire was compared against four or more pages did effects on response rate emerge [16].

We are aware of only one previous experimental study in a similar population of drinkers thinking about quitting or reducing their consumption that was not included in previous reviews [17]. It found that a brief alcohol consumption measure yielded a much higher response rate (51%) than did a more detailed and relatively time-consuming measure (22%). Both are commonly used approaches, though the time commitments involved have not been studied.

There are two previous online studies of the effects of questionnaire length on response rate. Both studies found shorter questionnaires to increase response rates by approximately 50% to 100%, which is in line with the mean size of effects observed in postal surveys. Deutskens and colleagues [18] compared a questionnaire taking approximately 15 to 30 minutes to complete with one taking 30 to 45 minutes to complete and found response rates of 24.5% and 17.1% respectively. Marcus and colleagues [19] compared a questionnaire with 91 items taking approximately 10 to 20 minutes to complete with one comprised of 359 items and described as taking 30 to 60 minutes to complete and obtained response rates of 30.8% and 18.6% respectively with the odds of response calculated as 0.51 (95% CI 0.42-0.62). Although the online follow-up context of the present study complicates a direct comparison with the wider literature, it seems very likely that the comparison we made is thus consistent with previous findings in that the difference between the two questionnaire lengths was simply too small to impact upon attrition.

### Relevance

Those participants asked more relevant questions in the form of items addressing alcohol problems rather than mental health were on average 3% less likely to be lost to follow-up. These additional questions followed detailed questions about recent alcohol consumption. These findings suggest that the perceived relevance of research assessments could indeed influence attrition.

Our emphasis here is on perceived relevance in the context of an alcohol rather than a mental health trial, even though the perception itself has not been directly assessed. Some participants undoubtedly did have mental health difficulties and may have seen the mental health instrument as being just as relevant to their situation as an alcohol problems measure had they been offered one. Study findings indicate that it is some unspecified property of this instrument that leads to lower follow-up rates in comparison with the others. We assumed at the outset, however, that across the study population as a whole, the mental health content of the additional questionnaire would be viewed as less relevant than an alcohol problems one, and this assumption formed the basis of the hypothesis and the operationalization of the relevance construct. This remains our interpretation of the characteristic most likely to be responsible for the observed difference, though the possibility must be recognized that other features may also be at work.

The existing literature on relevance is rather less extensive than that available for questionnaire length, though again observed effects are much larger than were observed here (unadjusted OR = 1.14, adjusted OR = 1.11). Relevance has also been operationalized heterogeneously in these previous studies. There were three postal studies included in the review by Edwards and colleagues [2] for which the combined odds of response were approximately doubled when more and less relevant questionnaires were compared (OR = 2.00, 95% CI 1.32-3.04). These studies compared the effects on response rates of questionnaires on (1) skipping classes among undergraduates versus PhD students [20], (2) providing bowling versus restaurant feedback among participants in an amateur bowling competition [21], and (3) “a variety of interesting topics” versus “ a boring topic in-depth” in market research [22] with the latter condition in each study being deemed to be less relevant. The only online relevance experiment of which we are aware found a similar effect size to ours (OR = 1.85, 95% CI 1.52-2.26) when comparing a highly *salient* questionnaire on the motives and personality of website owners against one on psychological aspects of Internet usage, which was deemed to be of relatively low salience among website owners [19]. As with the questionnaire length study, although comparisons are necessarily indirect, our relevance experiment involves a much less pronounced contrast than any previously studied, including this online experiment. We compared two questionnaires both judged relevant to the needs of the study population at the outset though differing in likely degree of relevance as perceived by study participants, whereas in the postal studies, the relevance experiments have been designed to compare relevant with not relevant.

### Putting the Findings Together

Our findings are strengthened by the large sample sizes employed, the randomized design, and the absence of any missing data given the nature of the study. The online context of the present study is important, as the Internet is likely to be the vehicle for an increasing number of studies of delivering health care and health promotion in the future, as well as many other types of research. The generalizability of data from this study population of hazardous and harmful drinkers to other populations is unknown.

The original decision to randomize to secondary outcome measures was influenced by the emerging literatures on “assessment reactivity” in the alcohol field [23-24] and on “mere measurement effects” elsewhere [25]. Work in this area suggests that participating in research studies and completing questionnaires can itself influence the target behavior under investigation, which, though relevant to all research designs, could be a particular problem in trials. The present findings on relevance, set in the context of the literature in this area, underscore how little is known about the unintended impacts of our research decisions on participant experience and behavior. The findings also indicate that attrition itself may be a useful proxy measure for unintended adverse effects of research design decisions.

We isolated two aspects of methodological decision making for experimental study here. Qualitative differences in questionnaire content were related to attrition, which suggests the possibilities that the aggregate effects of our methodological decisions may have a large influence not only on attrition but probably also on participant engagement with research in other ways. The absence of an effect of additional questionnaire length on attrition suggests that not all our decisions will do so. This suggestion is coherent with existing online findings of interactions between characteristics affecting response rates in surveys [19].

Further methodological studies of this type are important to pursue specifically in the context of both online and conventional trials and also more broadly, as the lack of prior study of the dynamics of response and attrition in different study designs should be rectified. Surely whether prospective research participants decide to enter studies or not, or stay in them if they do, depends upon what it is that is being asked of them.

## Abbreviations

APQ: Alcohol Problems Questionnaire
AUDIT: Alcohol Use Disorders Identification Test
CI: confidence interval
CORE-OM: Clinical Outcomes in Routine Evaluation-Outcome Measure
CORE-10: Clinical Outcomes in Routine Evaluation-10-item measure
DYD-RCT: Down Your Drink randomized controlled trial
EQ-5D: trade mark of the EuroQol group see http://www.euroqol.org/
LDQ: Leeds Dependence Questionnaire
